# Case Report: T-Dxd plus bevacizumab in a patient with brain metastases from rectal cancer with HER2 amplification

**DOI:** 10.3389/fonc.2025.1714422

**Published:** 2025-12-03

**Authors:** Yanan Jia, Xueqin Li, Huisheng Chen, Lisha Wu, Yinduo Zeng, Tao Qin

**Affiliations:** 1 Department of Medical Oncology, Shenshan Medical Center, Sun Yat-sen Memorial Hospital, Sun Yat-sen University, Shanwei, China; 2 Department of Medical Oncology, Sun Yat-sen Memorial Hospital, Sun Yat-sen University, Guangzhou, China; 3 Breast Tumor Center, Sun Yat-sen Memorial Hospital, Sun Yat-sen University, Guangzhou, China

**Keywords:** colorectal cancer, brain metastasis, HER2, T-DXd, bevacizumab

## Abstract

Brain metastasis from colorectal cancer was more common in patients with HER2 amplification. Here, we document a 53-year-old male with HER2-amplified rectal cancer and brain metastases, treated with Trastuzumab Deruxtecan (T-Dxd, 5.4 mg/kg) plus bevacizumab (7.5 mg/kg) every three weeks as third line therapy. The patient achieved a partial response with a progression-free-survival of 7 months, highlighting the potential of T-Dxd. Adverse events included grade 3 thrombocytopenia, grade 2 neutropenia, and grade 1 fatigue. Our report emphasizes the rarity of brain metastases in colorectal cancer and illustrates a favorable response to T-Dxd plus bevacizumab.

## Introduction

Globally, colorectal cancer (CRC) is the third most common cancer and the second leading cause of cancer-related mortality. Its incidence and mortality are rising in China (1, 2). Brain metastases from CRC are relatively rare, occurring in 0.6% to 3.2% of cases, with a median overall survival of only 5.5 months (3). These statistics underscore the urgent need for more effective treatment options. Current therapies for metastatic CRC are guided by molecular biomarkers, including mismatch repair and microsatellite instability, as well as HER2, KRAS, and BRAF mutation statuses. HER2, a receptor tyrosine kinase, plays a crucial role in tumorigenesis by activating downstream PI3K-AKT and MEK-ERK signaling pathways. HER2 amplification or overexpression occurs in approximately 3%-5% of metastatic CRCs (3, 4). Despite advances in targeted therapy, there are few effective options for HER2-positive rectal cancer with brain metastases. In this report, we describe a case of rectal cancer with HER2 amplification treated with T-Dxd and bevacizumab, focusing on its efficacy in addressing brain metastases.

## Case presentation

A 53-year-old man with an ECOG performance status of 0 had no prior medical conditions but had a family history of rectal cancer in his father and gallbladder cancer in his mother. A routine physical examination revealed an elevated serum carcinoembryonic antigen (CEA) level of 122.4 ng/mL (normal range: 0–5.0 ng/mL), and colonoscopy identified a 2 cm rectal mass. Pathological analysis showed severe atypical hyperplasia with glandular epithelial cancerization. PET-CT findings indicated multiple liver metastases. Next-generation sequencing (NGS, detecting instrument MGISEQ-2000, coverage 30 genes of gastrointestinal tumors, analysis pipeline Read1 Q30, amplification cutoff 1%) revealed microsatellite stability, HER2 amplification (copy number: 14.3), TP53 p.D57* (variant allele frequency (VAF) 14.5%), ERBB2 p.A270S (VAF 95.9%), and wild-type KRAS, NRAS, and BRAF. The disease was staged as rectal cancer cT3N2M1.

**Figure 1:**
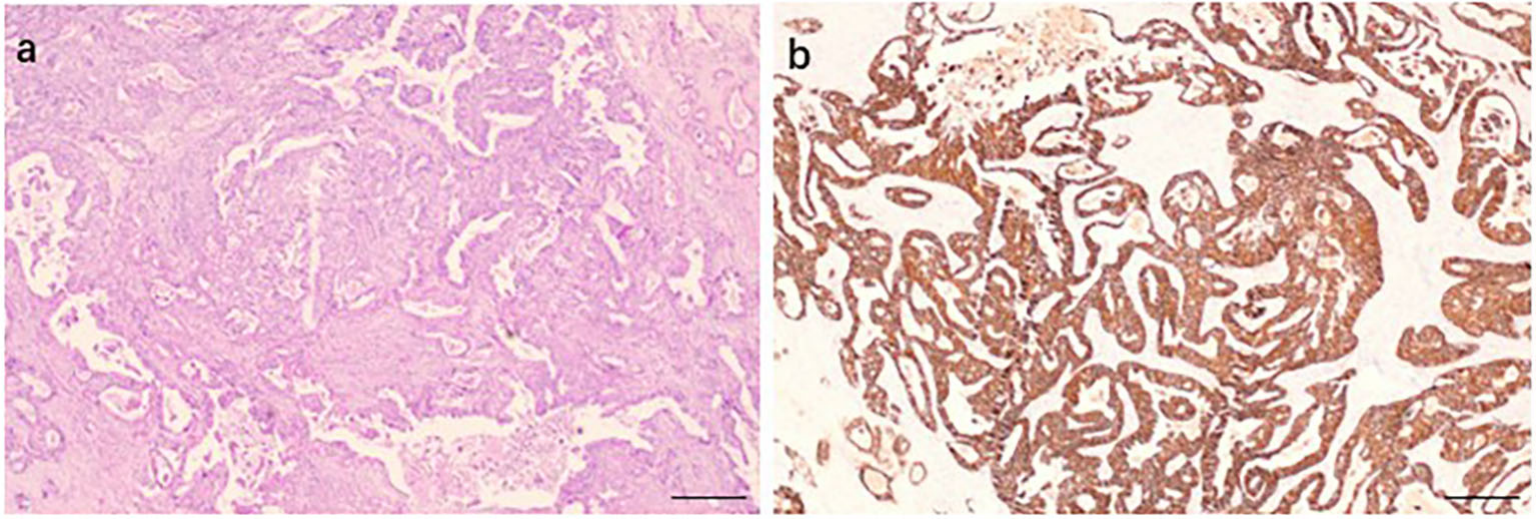
Hematoxylin and eosin **(a)** and HER2 immunohistochemical staining **(b)** of the primary rectal tumor (IHC 3 plus, magnification ×200, scale bar 100 micrometers).

The patient received initial therapy with bevacizumab, leucovorin, fluorouracil, oxaliplatin, and irinotecan (FOLFOXIRI + bevacizumab) for seven cycles. The best response was a partial response (PR) in liver metastases, with significant shrinkage per RECIST version 1.1 criteria. In March 2022, the patient underwent primary tumor resection, with pathology revealing T3 moderately differentiated adenocarcinoma and metastases in 5 of 25 dissected lymph nodes. Ki-67 expression was 60% and HER2 was positive (**Figure 1**). Meanwhile, there was no mismatch repair deficiency. Post-surgery, the patient completed five additional cycles of FOLFOXIRI plus bevacizumab, with the last chemotherapy administered in August 2022.

**Figure 2:**
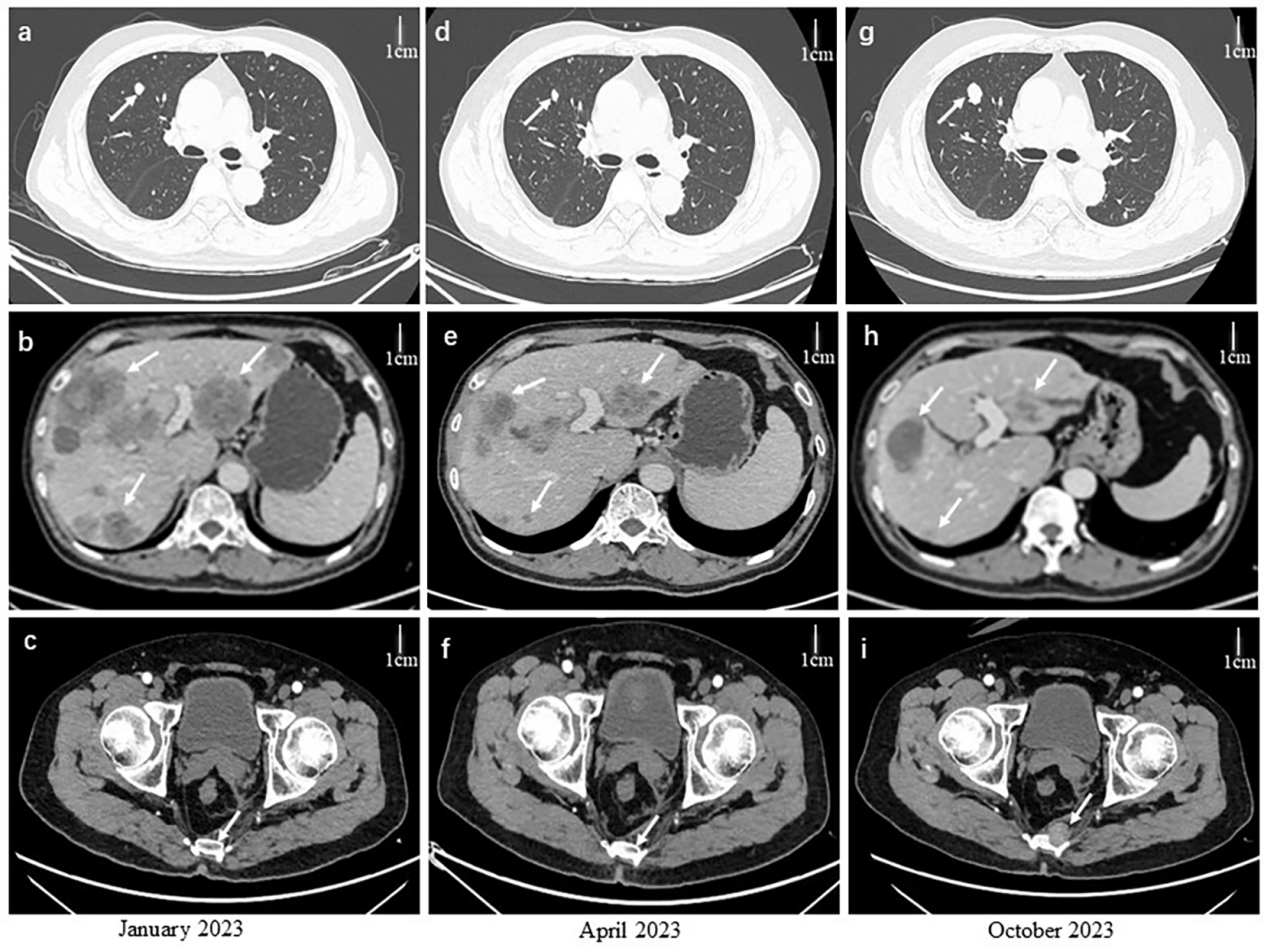
Arrows indicate tumor location. CT in January 2023 (after first line therapy) showed progression **(a–c)**. April 2023 scans showed significant regression **(d–f)**. October 2023 scans showed progression after trastuzumab plus pertuzumab with mXELIRI **(g–i)**. Scale bars represent one centimeter.

In January 2023, serum levels of CEA and carbohydrate antigen 19-9 (CA19-9) rose significantly to 5645.75 ng/mL and >1200 U/mL (normal range: 0–43 U/mL), respectively. CT scans showed recurrent rectal tumor, increased liver metastases, and new lung metastases. Based on genomic testing results, a regimen of trastuzumab and pertuzumab combined with mXELIRI (capecitabine 800 mg/m² twice daily for two weeks, irinotecan 200 mg/m² every three weeks) was administered from February 5 to September 2023 (12 cycles). The best response was a PR (**Figure 2**), with CEA and CA19–9 levels initially decreasing to 272 ng/mL and 826 U/mL before rising again to 349 ng/mL and 1434 U/mL. The main adverse event during treatment was grade 2 thrombocytopenia, which occurred without bleeding.

**Figure 3:**
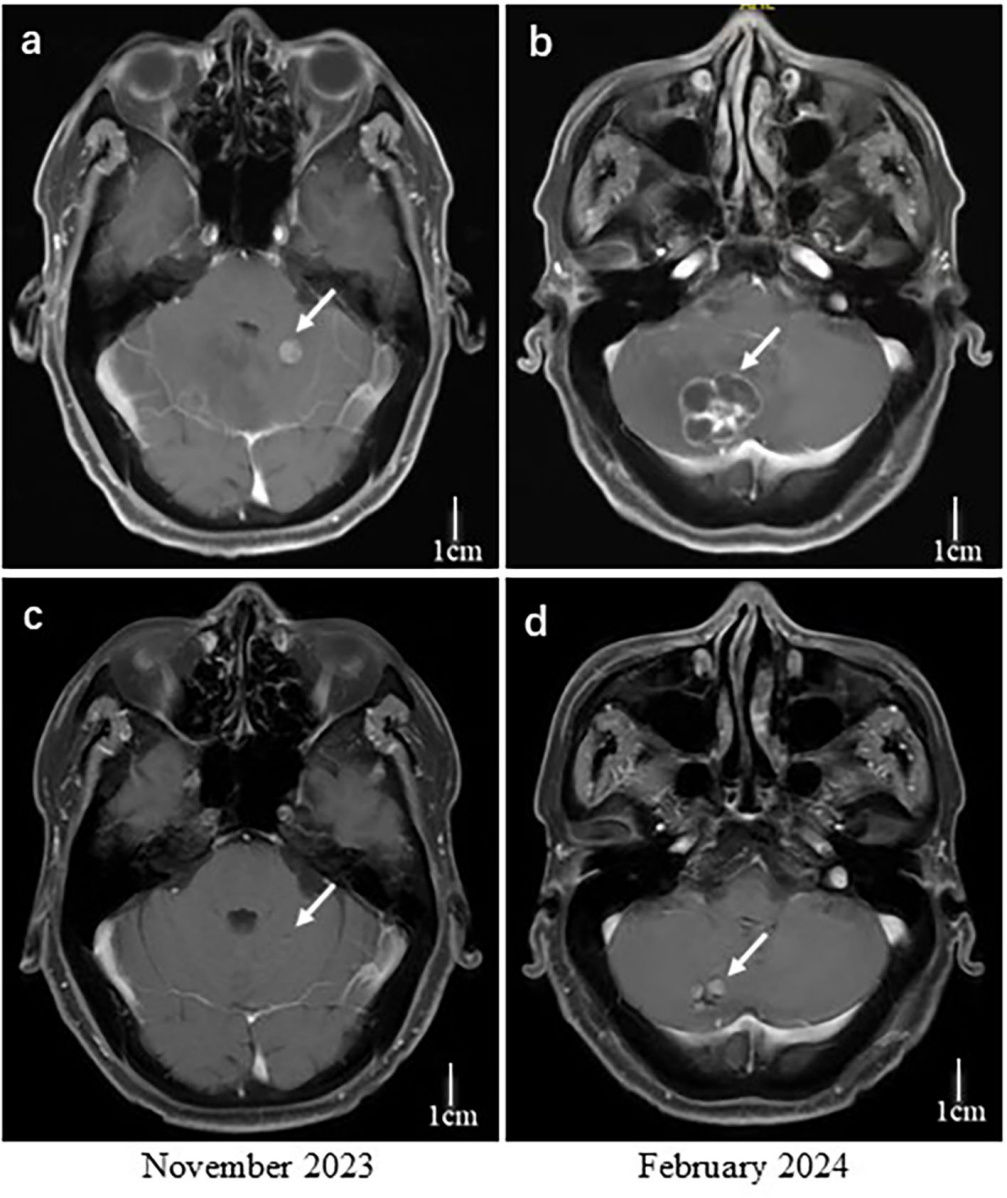
Contrast-enhanced T1-weighted MRI images of the brain before treatment **(a, b)** and after four cycles of T-Dxd plus bevacizumab **(c, d)**. Scale bars represent one centimeter.

Subsequently, the patient developed new-onset headaches, and imaging showed progression of rectal and lung tumors along with multiple new brain metastases in the cerebellum. In October 2023, Trastuzumab deruxtecan (T-Dxd) was started at 5.4 mg/kg every three weeks combined with bevacizumab 7.5 mg/kg every three weeks as third-line therapy. After four cycles, the best response was a PR in the central nervous system (CNS) metastases (**Figure 3**). Stereotactic body radiation therapy (SBRT) was administered after PR using a dose of 30 Gy in five fractions to the cerebellar lesions. Adverse events included grade 3 thrombocytopenia, grade 2 neutropenia, and grade 1 fatigue. No interstitial lung disease was observed on follow-up chest imaging. In May 2024, PET-CT showed disease progression of lung and sacrum metastases. The duration of response for T-Dxd treatment was approximately 7 months.

**Figure 4:**
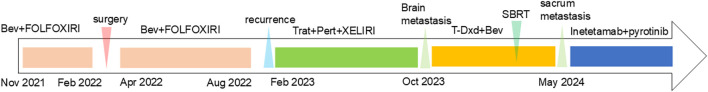
The treatment timeline of this patient. Bev, bevacizumab; Trat, Trastuzumab; Pert, Pertuzumab; SBRT, Stereotactic body radiation therapy.

No further tissue biopsy or genetic testing was conducted due to the patient’s condition after the tumor progressed. From May to November 2024, the patient received pyrotinib 400 mg daily with inetetamab 8 mg/kg loading dose followed by 6 mg/kg every three weeks, achieving stable disease. At the last follow-up in Jan 2025, the patient remained alive with good performance status and an overall survival exceeding 36 months. The treatment timeline for the patient is shown in **Figure 4**.

## Discussion

Our study underscores the promising efficacy of T-Dxd and bevacizumab in the treatment of HER2-amplified rectal cancer with brain metastases, demonstrating significant tumor shrinkage consistent with partial remission. The patient achieved a disease control duration of 7 months and an overall survival (OS) of 36 months.

The incidence of colorectal cancer is rising, which is one of most common malignant tumors in China (5). For metastatic rectal cancer, targeted therapy tailored to RAS and microsatellite status, along with HER2 testing, is strongly recommended after chemotherapy includes regimens incorporating oxaliplatin or irinotecan. The PETACC-3 study described molecular differences between distal and proximal colon cancers but did not report HER2 at such high frequencies. HER2 amplification in metastatic CRC occurs in about 3%-5% of cases and is enriched in left-sided RAS-wild-type tumors (6). Optional anti-HER2 therapies include trastuzumab, pertuzumab, tucatinib, trastuzumab emtansine (T-DM1), T-Dxd, and RC48 (7). In the MyPathway study, trastuzumab plus pertuzumab achieved an overall response rate (ORR) of 32%, median progression-free survival (PFS) of 5.3 months, and OS of 10.9 months. In the TAPUR study, the ORR was 25% with median PFS about 17 weeks, and in TRIUMPH the ORR was 28% to 30% (8–10).

Several studies reported that HER2 has been linked to high probability of brain metastases, less sensitivity of anti-EGFR therapy and poorer prognosis (11–14). Although brain metastases are relatively rare in CRC, their incidence has gradually increased with an incidence of 2% patients were diagnosed with brain metastases (15). A prior study reported HER2 amplification in 18.4% (16 of 87) of CRC cases with brain metastases, demonstrating a significantly higher risk compared to HER2-negative CRC cases, with accounted to 75% concordance rate in HER2 status between primary CRC tumors and paired brain metastases sample (16, 17). At present, the mechanism of ADC acting on brain metastases is unclear, possible mechanisms were as follows: (1)During the process of brain metastasis, the blood-brain barrier(BBB) transforms into the blood-tumor barrier, resulting in increased permeability (18); (2)Radiotherapy can further damage the BBB, thereby enabling large molecule drugs to pass through it (19); (3)”By-stander killing” may be a reason, which could benefit not only antigen-expressing tumor cells but also adjacent antigen-negative cells through the transfer of released payload, which may enter brain metastases, from the antigen-expressing cells to the neighboring antigen-negative cells (20). However, limited data to once brain metastases were reported, MDT was recommended because prognosis was poor. Surgical rection and radiotherapy surgery were important options for those limited metastases.

Besides, Bevacizumab, an anti-VEGF monoclonal antibody, may improve survival outcomes in patients with CRC and brain metastases by inhibiting tumor angiogenesis and reducing cerebral edema. Although its ability to penetrate the BBB is limited, combination with chemotherapy or radiotherapy can enhance local control efficacy. Bevacizumab combined with chemotherapy in colorectal cancer patients with brain metastases achieved a median OS of 20.6 months after the diagnosis of brain metastases (21). Furthermore, bevacizumab may enhance antibody-drug conjugate delivery by normalizing tumor vasculature and reducing edema, although this synergy remains hypothetical in colorectal cancer.

As to systemic therapy targeting the HER2 pathway, the dual-targeted combination of trastuzumab and pertuzumab has shown enhanced intracranial control in HER2-positive colorectal cancer. In the HERACLES-A phase II trial, 914 RAS-wild-type mCRC cases were screened and 48 were HER2-positive. Among 27 treated patients, trastuzumab plus lapatinib produced an ORR of 30%, disease control rate of 74%, and median PFS of 5.2 months (22). The MyPathway basket trial further validated the potential of dual-antibody therapy, reporting an ORR of 38% in the HER2-amplified colorectal cancer cohort treated with trastuzumab plus pertuzumab, alongside median PFS and OS of 5.3 months and 10.9 months, respectively, with partial intracranial lesion responses observed in some cases (8). Although existing data support the clinical benefits of combination therapies, evidence primarily originates from phase II trials or retrospective studies, underscoring the need for larger dedicated studies focusing on brain metastases to validate these findings. In addition, the clinical activity of T-Dxd was promising in patients with brain metastases of HER2 positive breast cancer (23). In DESTINY-CRC01 cohort A (HER2 IHC3 plus or IHC2 plus and ISH positive), the ORR was 45.3%, while cohorts B and C had 0% responses. In DESTINY-CRC02, the ORR was 37.8% with 5.4 mg/kg and 27.5% with 6.4 mg/kg, with interstitial lung disease rates of 8% and 13% respectively. Median PFS was 5.8 to 6.9 months, and a median OS was 13.4 to 15.5 months after T-Dxd (24–26). The above studies indicated that T-Dxd could control disease both extracranial and intracranial metastases. Our case benefit from T-Dxd showed good response and long disease control duration. A recent study advanced these findings by employing single-cell analysis to map the immune microenvironment of human melanoma brain metastases under immune checkpoint inhibition, revealing dynamic cellular interactions (27). We anticipate the results of the ongoing MOUNTAINEER-03 phase III trial, which is evaluating tucatinib combined with trastuzumab and chemotherapy as first-line treatment for HER2-positive metastatic colorectal cancer (28). Furthermore, there are limitations of our study noting that this is a single-patient report, that SBRT was delivered concurrently, and that attribution of benefit to T-Dxd versus radiation is uncertain.

Given these promising results and the favorable tolerability of anti-HER2 therapies, routine evaluation of HER2 expression in patients with advanced colorectal cancer is strongly recommended. This evolution has the potential to advance personalized medicine, enabling treatment strategies that not only enhance survival outcomes but also improve the quality of life for patients facing this challenging disease.

## Data Availability

The original contributions presented in the study are included in the article/supplementary material. Further inquiries can be directed to the corresponding author/s.
